# Ambulatory Toxicity Management (AToM) Pilot: results of a pilot study of a pro-active, telephone-based intervention to improve toxicity management during chemotherapy for breast cancer

**DOI:** 10.1186/s40814-019-0404-y

**Published:** 2019-03-08

**Authors:** Monika K. Krzyzanowska, Cassandra MacKay, Heekyung Han, Maria Eberg, Sonal Gandhi, Nicole B. Laferriere, Melanie Powis, Doris Howell, Clare L. Atzema, Kelvin K. W. Chan, Vishal Kukreti, Sandra Mitchell, Marla Nayer, Mark Pasetka, Dafna Knittel-Keren, Erin Redwood

**Affiliations:** 1 Cancer Care Ontario, Toronto, ON Canada; 2 University Health Network, Toronto, ON Canada; 3 Sunnybrook Regional Health Sciences Centre, Toronto, ON Canada; 4 Thunder Bay Regional Health Sciences Centre, Thunder Bay, ON Canada; 5 Canadian Centre for Applied Research in Cancer Control, Toronto, ON Canada; 6 National Cancer Institute, Bethesda, MD USA; 7 University of Toronto, Toronto, ON Canada

**Keywords:** Breast cancer, Symptom management, Chemotherapy toxicity, Telephone case management, Quality improvement

## Abstract

**Background:**

Chemotherapy is associated with a significant risk of toxicity, which often peaks between ambulatory visits to the cancer centre. Remote symptom management support is a tool to optimize self-management and healthcare utilization, including emergency department visits and hospitalizations (ED+H) during chemotherapy. We performed a single-arm pilot study to evaluate the feasibility, acceptability, and potential impact of a telephone symptom management intervention on healthcare utilization during chemotherapy for early stage breast cancer (EBC).

**Methods:**

Women starting adjuvant or neoadjuvant chemotherapy for EBC at two cancer centres in Ontario, Canada, received standardized, nurse-led calls to assess common toxicities at two time points following each chemotherapy administration. Feasibility outcomes included patient enrollment, retention, RN adherence to delivering calls per the study schedule, and resource use associated with calls; acceptability was evaluated based on patient and provider feedback. Impact on acute care utilization was evaluated post hoc by linking individual patient records to provincial data holdings to examine ED+H patterns among participating patients compared to contemporaneous controls.

**Results:**

Between September 2013 and December 2014, 77 women were enrolled (mean age 55 years). Most commonly used regimens were AC-paclitaxel (58%) and FEC-docetaxel (16%); 78% of patients received primary granulocyte colony-stimulating factor prophylaxis. 83.8% of calls were delivered per schedule; mean call duration was 9 min. The intervention was well received by both patients and clinicians. Comparison of ED+H rates among study participants versus controls showed that there were fewer ED visits in intervention patients [incidence rate ratio (IRR) (95% CI) = 0.54 (0.36, 0.81)] but no difference in the rate of hospitalizations [IRR (95% CI) = 1.02 (0.59, 1.77)]. Main implementation challenges included identifying eligible patients, fitting the calls into existing clinical responsibilities, and effective communication to the patient’s clinical team.

**Conclusions:**

Telephone-based pro-active toxicity management during chemotherapy is feasible, perceived as valuable by clinicians and patients, and may be associated with lower rates of acute care use. However, attention must be paid to workflow issues for scalability. Larger scale evaluation of this approach is in progress.

**Electronic supplementary material:**

The online version of this article (10.1186/s40814-019-0404-y) contains supplementary material, which is available to authorized users.

## Background

Systemic therapy can improve the outcomes of patients with cancer, but carries a substantial risk of toxicity, which often peaks between visits to the cancer clinic. Suboptimal management of toxicities can lead to emergency department visits and hospitalizations (ED+H) during treatment. The growing number of studies reporting high rates of acute care utilization during chemotherapy across countries and settings [1, 2] suggests that our current models for managing toxicities among patients receiving chemotherapy in ambulatory settings may be inadequate. By providing patients with self-management strategies or alternatives to the emergency department for further assessment, early intervention can help to address toxicities before they become too severe [3, 4].

Pro-active management of symptoms has been shown to decrease acute care use in chronic diseases such as congestive heart failure [5]. In oncology specifically, studies suggest that remote, often telephone-based, nurse-led symptom-focused interventions are feasible, may improve symptom control and decrease unscheduled emergency room visits and hospitalizations [6–11]. A recently published, single-centre randomized controlled trial of remote web-based patient-reported symptom assessments coupled with automatic provider alerts [12, 13] demonstrated that patients receiving remote monitoring had fewer emergency department visits (34% vs 41%) or hospitalizations (45% vs 49%), remained on chemotherapy longer (8.2 vs 6.3 months), and had better survival relative to patients receiving usual follow-up care. All of these studies, however, have evaluated the impact of these types of interventions in the research setting and have not addressed the additional barriers associated with the implementation and sustainability in routine ambulatory cancer care. As such, further evaluation is needed to inform effective integration of pro-active toxicity management into existing models of care.

The purpose of this single-arm pilot study was to assess the feasibility and acceptability of implementing a nurse-led, pro-active, telephone-based toxicity management into routine clinical practice for patients receiving chemotherapy for early-stage breast cancer and to evaluate potential impact on emergency room visits and hospitalizations during treatment. The goal was to inform larger scale implementation and evaluation. We chose to focus on women with breast cancer as previous population-based studies have reported high rates of treatment-related toxicities and acute care utilization during treatment in this patient population [14–16]. In addition, confounding from symptoms related to advanced cancer is likely to be small in this population given the early stage of disease.

## Methods

### Study design and participants

To evaluate the feasibility and acceptability of implementing a pro-active, telephone-based toxicity management intervention, we undertook a single-arm prospective pilot study in two academic institutions with integrated cancer programs, in Ontario, Canada, that responded to a request to participate. Women newly diagnosed with early-stage breast cancer (stages I–III) who were initiating adjuvant or neoadjuvant chemotherapy, had an adequate command of English to complete questionnaires, and provided individual consent to participate were eligible. Patients receiving treatment with an investigational agent were excluded because the focus of the intervention was on patients treated in routine care. To understand the feasibility of using administrative data to look at acute care utilization during treatment and evaluate the potential impact of the intervention on acute care utilization, an administrative data-based analysis was also undertaken. A contemporaneous control cohort was identified after the study completion from the Activity Level Report (ALR) database, which was linked to other administrative databases to capture information on patient demographic and clinical characteristics, and outcomes. The control cohort consisted of all other patients diagnosed with early-stage breast cancer who were initiating the same chemotherapy regimens as study participants in the two participating institutions during the study intervention period but who did not participate in the prospective pilot (Additional file 1: Figure S1).

### Description of the intervention

Educational handouts discussing treatments and management of toxicities dispensed to patients initiating treatment differ by provider and cancer centre. As such, all patients were provided with a standardized symptom self-management guide prior to initiating chemotherapy which included recommendations for self-management of common toxicities. The content of the guide was adapted from information available on the Canadian Cancer Society and American Cancer Society web pages. The guide covered a subset of 9 toxicities from the National Cancer Institute’s Patient-Reported Outcomes version of the Common Terminology Criteria for Adverse Events (NCI PRO-CTCAE). These were chosen from the 124-item bank through expert consensus (by the project steering committee) to reflect commonly experienced toxicities by breast cancer patients who are treated with chemotherapy that may lead to ED+H and are amenable to early intervention [17, 18]. The subset of toxicities included the following: nausea, vomiting, mouth and throat sores, pain, aching joints and muscles, loose and watery stools, shivering or shaking chills, constipation, and fatigue.

**Fig. 1:**
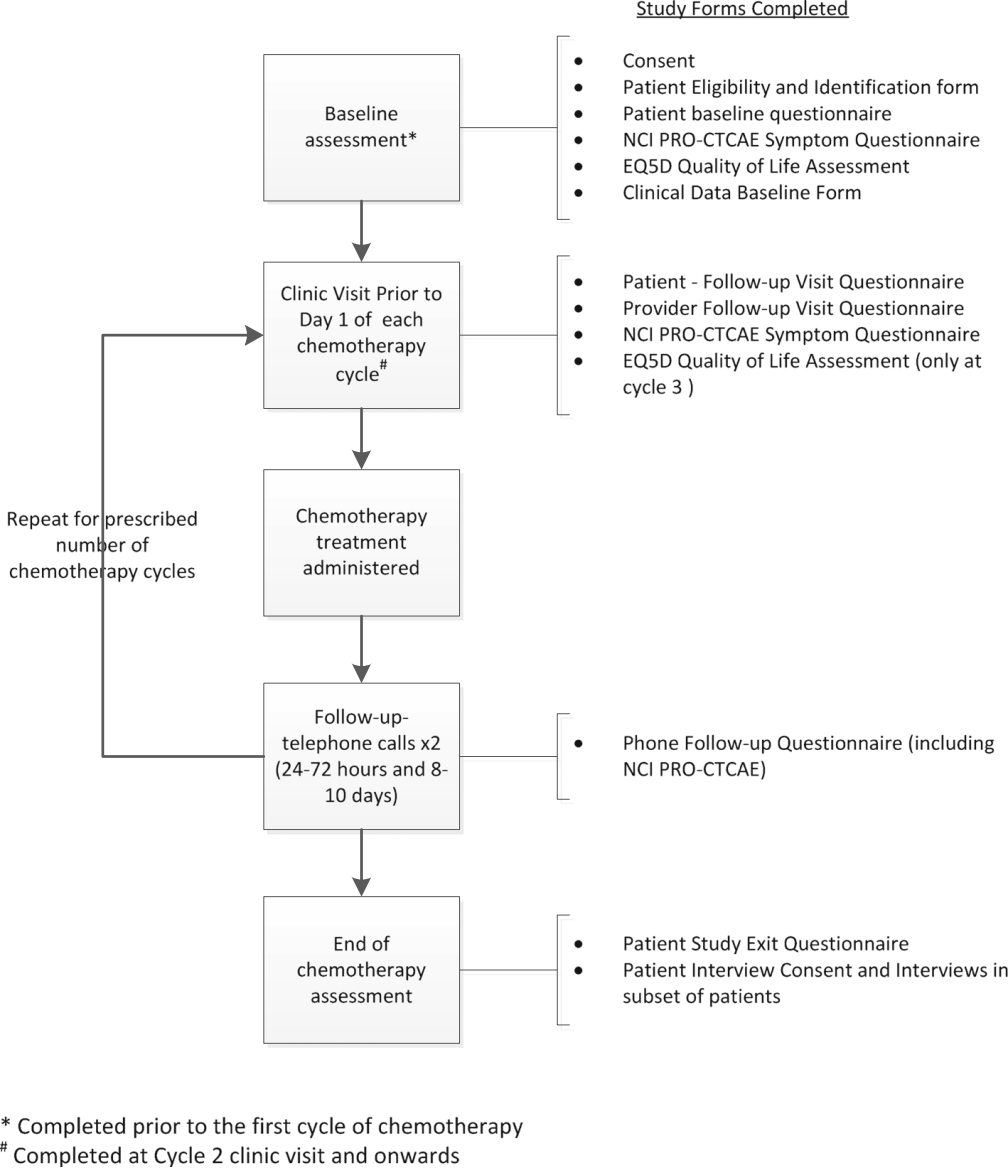
Schedule of study activities and assessments

The intervention consisted of two pro-active telephone calls following each chemotherapy administration: at 24 to 72 h and at 8 to 10 days post-chemotherapy (Fig. 1). The calls were made by nurses located at the patient’s treating institution. Participating nurses received training on the study protocol and intervention during a study kick-off meeting and participated in monthly study calls to troubleshoot logistics of delivering the intervention. Toxicity assessments, management recommendations, and documentation were standardized using a form covering the same nine toxicities as the patient symptom self-management guide and a companion provider symptom management guide, consistent with best practices and current evidence. Local implementation logistics regarding delivery of the intervention and data collection were determined by the participating centres.

### Analysis of feasibility and acceptability

The primary focus of the evaluation was on the feasibility and acceptability of implementation, for which a target recruitment of up to 100 women was considered sufficient based on the literature [19, 20]. A formal sample size calculation was not undertaken. Feasibility was assessed based on patient enrollment, retention, RN adherence to delivering calls per the study schedule, and resource use (non-pharmacological or pharmacological recommendations, healthcare provider visit or urgent care). Acceptability of the intervention was assessed from the perspectives of patients and providers using end-of-study surveys (patients) and semi-structured interviews (patients and providers). Following the completion of the last cycle of chemotherapy and related calls, all patients were invited to complete an end-of-study survey that sought feedback on the patient symptom self-management guide and the follow-up calls. Feasibility and acceptability findings are reported as a proportion; 95% confidence intervals (95% CI) were calculated to represent variance in the observed proportion. A convenience sample of 17 patients also participated in the end-of-study semi-structured telephone interviews on the patients’ experiences with the intervention. In addition, interviews were conducted with providers involved in delivering the intervention to evaluate the barriers and facilitators of implementation and recommendations for improving intervention delivery. Qualitative data from the interviews was analysed using inductive content analysis to derive themes and sub-themes that were grounded in the experience of patients and clinicians [21]. Whether or not the intervention was considered acceptable and feasible was determined by the project steering committee based on the review of the experience with recruitment, delivery of intervention, and feedback from patients and providers following completion of the study.

### Analysis of acute care utilization

An administrative data-based analysis was undertaken to assess the feasibility of using administrative data to look at ED+H, to evaluate sample representativeness, and to compare the rates of acute care visits among pilot study participants against patients receiving care at the participating centres during the same time frame but who did not participate in the pilot (contemporaneous controls). Pilot study participants were identified deterministically in the provincial data holdings. Information on chemotherapy regimen was obtained from the Activity Level Reporting (ALR) database. Information on ED visits and hospitalizations from initiation of chemotherapy until 30 days after the final dose was obtained from the National Ambulatory Care System (NACRS) database and Canadian Institute for Health Information Discharge Abstract Database (CIHI DAD), respectively. Acute care visits were classified as ED visit only or a hospitalization, defined as having experienced either a direct admission or an ED visit resulting in an admission. Demographic and clinical characteristics of the intervention and control cohorts were summarized using descriptive statistics. Standardized differences were assessed between the patients receiving the intervention and the controls. Negative binomial regression with an offset for time was used to estimate the relationship between event rates and the study intervention, overall and stratified by centre. We undertook a stratified analysis by centre due to the differences in regimen, patient characteristics such as cancer stage and age, baseline rates of acute care utilization, and centre characteristics such as rurality which may affect the way patients would seek and receive medical care but could not be adjusted for due to small sample size. We used clinical expertise and statistical evaluation to select the most important variables for adjustment in regression models.

## Results

### Feasibility

Between September 2013 and December 2014, 77 patients were accrued at the 2 participating cancer centres; recruitment was slower than anticipated at both sites. At centre 1, recruitment was initially limited to 2 of the 7 breast clinics and occurred in batches of 10 patients at a time, in order to facilitate the workload of the intervention nurse (who was also a clinical trials nurse). Later, it was expanded to patients from all 7 clinics. At centre 2, initial patient recruitment was carried out by clinic staff and calls were made by a senior clinic nurse with expertise in symptom management. Recruitment was slow due to the lack of a standardized approach to identify eligible patients since this was a new task for the clinic staff and the lack of ethics training by 1 of the physicians in the clinic which made their patients ineligible for recruitment. However, once the local research staff were enlisted to facilitate patient identification, recruitment improved at the centre. Of the 77 patients enrolled, 75 completed the intervention; 1 patient withdrew due to the progression of another illness and 1 died during treatment secondary to sepsis. Demographics and clinical characteristics of the patients are summarized in Table 1.

**Table 1:** Baseline demographic and clinical characteristics of study participants

Variable	Centre 1, *n* = 56	Centre 2, *n* = 21	Total, *n* = 77
Age, mean (SD)	53.8 (10.9)	58.7 (11.1)	55.1 (11.1)
Married, *n* (%)	38 (67.9)	16 (76.2)	54 (70.1)
Highest level of education, *n* (%)			
Less than college/university	11 (19.6)	4 (19.0)	15 (19.5)
College/university or higher	39 (69.6)	17 (81.0)	66 (85.7)
Prefer not to respond	6 (10.7)	0	6 (7.8)
Combined household income, *n* (%)			
< $60 k	19 (33.9)	5 (25.0)	24 (31.2)
$60–99 k	10 (17.9)	5 (25.0)	15 (19.5)
> 100 k	11 (19.6)	5 (25.0)	16 (21.1)
Prefer not to respond	16 (28.6)	5 (25.0)	21 (27.6)
Employment status, *n* (%)			
Employed (working or on sick leave)	31 (55.4)	10 (47.6)	41 (53.2)
Unemployed or retired	20 (35.7)	10 (47.6)	30 (39.0)
Stage, *n* (%)			
Stage I	6 (10.7)	3 (15.0)	9 (11.8)
Stage II	25 (44.6)	5 (25.0)	30 (39.5)
Stage III	25 (44.6)	12 (60.0)	37 (48.7)
Treatment intent, *n* (%)			
Adjuvant	42 (75.0)	14 (70.0)	56 (73.7)
Neoadjuvant	14 (25.0)	6 (30.0)	20 (26.3)
Regimen, *n* (%)			
AC-P	30 (53.6)	15 (60.0)	45 (58.4)
FEC-100	3 (5.4)	5 (25.0)	8 (10.5)
FEC-T	12 (21.4)	0	12 (15.6)
TC	10 (17.9)	0	10 (13.2)
Other	1 (1.8)	1 (5.0)	2 (2.6)
Primary G-CSF prophylaxis, *n* (%)	50 (89.2)	9 (42.9)	59 (76.6)
Central line, *n* (%)	20 (35.7)	5 (23.8)	25 (32.5)
Co-morbidities, *n* (%)			
Cardiovascular disease	3 (5.4)	2 (9.5)	5 (6.5)
Chronic lung disease	3 (5.4)	1 (4.8)	4 (5.2)
Diabetes	4 (7.1)	2 (9.5)	6 (7.8)
Moderate to severe kidney disease	1 (1.8)	1 (4.8)	2 (2.6)

*SD* standard deviation; *AC-P* adriamycin, cyclophosphamide, and paclitaxel; *FEC-100* 5-fluorouracil, epirubicin, and cyclophosphamide; *FECT-T* 5-fluorouracil, epirubicin, cyclophosphamide, and docetaxel; *TC* docetaxel and cyclophosphamide; *G-CSF* granulocyte colony-stimulating factor

**Fig. 2:**
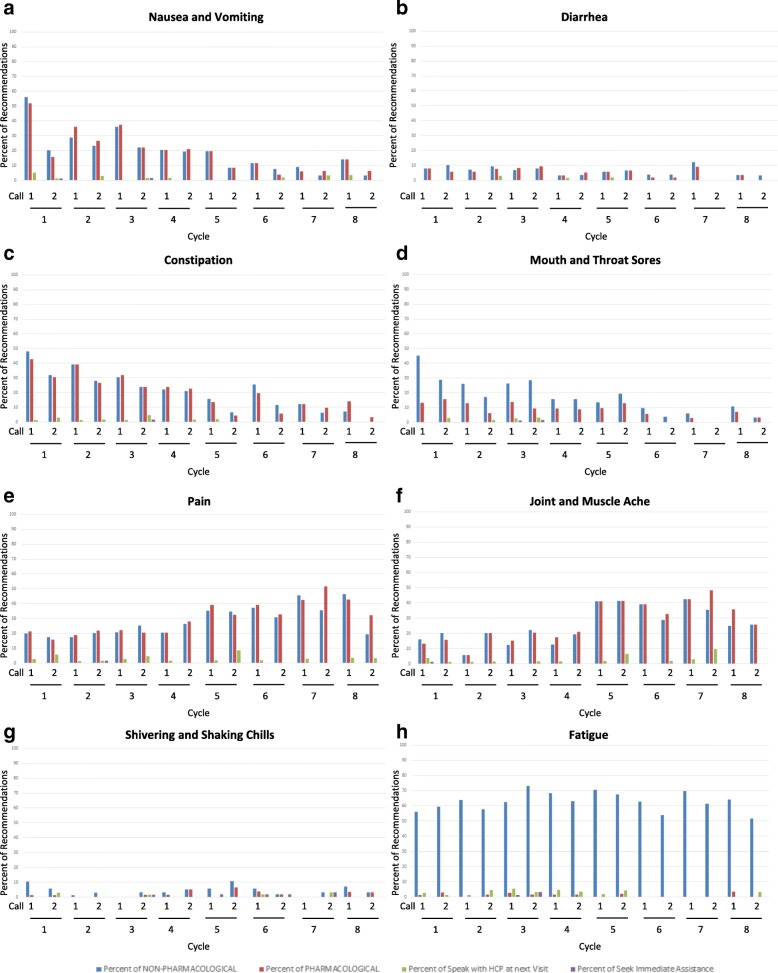
Types of recommendations made during follow-up calls by cycle and symptom for nausea and vomiting (**a**), diarrhea (**b**), constipation (**c**), mouth and throat sore (**d**), pain (**e**), joint and muscle ache (**f**), shivering and shaking chills (**g**), and fatigue (**h**)

83.8% (95% CI 81.4–86.0%) of expected calls were delivered per the study schedule. During the course of the intervention, 855 pro-active calls were delivered, with a mean duration of 9 min. The type of symptoms encountered varied by treatment cycle. Symptoms such as nausea, vomiting, and constipation required intervention in early cycles, whereas pain and joint and muscle aches were more prevalent in later cycles, often after the initiation of the taxane-based portion of the regimen. Of the 3131 recommendations made, 56.9% (95% CI 55.2–58.7%) were non-pharmacological, 35.1% (95% CI 33.4–36.8%) were pharmacological, 7.1% (95% CI 6.2–8.0%) required follow-up at the next clinic visit, and 0.9% (95% CI 0.6–1.3%) were to seek immediate assistance. For most symptoms, there was a roughly equal split in non-pharmacologic and pharmacologic interventions with the exception of fatigue where recommendations were mostly non-pharmacologically focused (Fig. 2).

### Acceptability

Among the 70 patients who completed the end-of-study survey, most reported that their symptoms were well controlled and that they used the self-management guide to manage symptoms during treatment (85.7%, 95% CI 75.3–92.9%; Table 2). The majority of patients liked receiving the calls (97.1%, 95% CI 90.1–99.6%) and would recommend that a similar program be extended to all patients receiving chemotherapy (94.3%, 95% CI 86.0–98.4%). In the end-of-study interviews, patients reported enjoying the relationship they developed with the nurse delivering the calls and that telephone support helped to normalize their experience and boosted their confidence for self-care by providing just-in-time education and support (Table 3).

**Table 2:** End-of-study feedback from participants by study site

Questions	Participant response	Centre 1		Centre 2		Total	
		*N* = 49 (%)	95% CI	N = 21 (%)	95% CI	*N* = 70 (%)	95% CI
NCI PRO-CTCAE tool							
The NCI PRO is important because it helps my healthcare team and research coordinator know what symptom I am having and how severe they are.	Strongly agree/somewhat agree	49 (100)	92.8–100	20 (95.2)	76.2–99.9	69 (98.6)	92.3–100
The symptom levels made it easier for me to describe how I am physically feeling.	Strongly agree/somewhat agree	47 (95.9)	86.0–99.5	20 (95.2)	76.2–99.9	67 (95.7)	88.0–99.1
Symptom management guide							
During your chemotherapy treatment, did you use the symptom management guide provided to you?	Yes	45 (91.8)	80.4–97.7	15 (71.4)	47.8–88.7	60 (85.7)	75.3–92.9
How often did you refer to the symptom management Guide? (number of times/chemotherapy cycle)	Never	4 (8.2)	2.3–19.6	6 (28.6)	11.3–52.2	10 (14.3)	7.1–24.7
	1–3	28 (57.1)	42.2–71.1	13 (61.9)	38.4–81.9	41 (58.6)	46.2–70.2
	Greater than 3 times	12 (24.5)	13.3–38.9	2 (9.5)	1.2–30.4	14 (20.0)	11.4–31.3
	Missing	5 (10.2)	3.4–22.2	0	0–16.1	5 (7.1)	2.4–15.9
Did you find the symptom management guide helpful in managing your symptoms related to chemotherapy?	Strongly agree/somewhat agree	44 (89.7)	77.8–96.6	14 (66.7)	43.0–85.4	58 (82.9)	72.0–90.8
Did you feel that the symptom management guide improved your ability to self-manage your chemotherapy side effects?	Strongly agree/somewhat agree	44 (89.7)	77.8–96.6	15 (71.4)	47.8–88.7	59 (84.3)	73.6–91.9
Did you feel that the symptom management guide helped you to understand when to seek medical care?	Strongly agree/somewhat agree	46 (93.9)	83.1–98.7	15 (71.4)	47.8–88.7	61 (87.1)	77.0–94.0
Pro-active, telephone-based symptom management calls							
Did you like receiving the follow-up phone calls?	Strongly agree/somewhat agree	49 (100)	92.8–100	19 (90.5)	69.6–98.8	68 (97.1)	90.1–99.7
Did you find the follow-up phone calls to be a burden?	Strongly agree/somewhat agree	2 (4.1)	0.5–14.0	2 (9.5)	1.2–30.4	4 (5.7)	1.6–14.0
Were the follow-up calls helpful in managing your symptoms?	Strongly agree/somewhat agree	47 (95.9)	86.1–99.5	17 (81.0)	58.1–94.6	64 (91.4)	82.3–96.8
Overall							
During treatment, my physical symptoms have been controlled to a comfortable level (examples of physical symptoms are nausea, pain, constipation, etc.)	Strongly agree/somewhat agree	48 (98.0)	89.2–100	20 (95.2)	76.2–99.9	68 (97.1)	90.1–99.7
During treatment, my emotional symptoms have been controlled to a comfortable level (examples of emotional symptoms are anxiety, depression, etc.)	Strongly agree/somewhat agree	48 (98.0)	89.2–100	16 (76.2)	52.8–91.8	64 (91.4)	82.3–96.8
Do you feel that participating in this study prevented you from going to the emergency room as a result of you chemotherapy side effects?	Strongly agree/somewhat agree	35 (71.4)	56.7–83.4	12 (57.1)	34.0–78.2	47 (67.1)	54.9–77.9
	Strongly disagree/somewhat disagree	8 (16.3)	7.3–29.7	5 (23.8)	8.2–47.2	13 (18.6)	10.3–29.7
Would you recommend this study protocol (symptom assessment, symptom management, and follow-up phone calls) be adapted to all cancer patients getting chemotherapy?	Strongly agree/somewhat agree	47 (95.9)	86.1–99.5	19 (90.5)	69.6–98.8	66 (94.3)	86.0–98.4

*CI* confidence interval, *NCI PRO CTCAE* National Cancer Institute Patient Reported Outcomes Common Terminology Criteria for Adverse Events

**Table 3:** Thematic analysis of end-of-study patient (*n* = 17) interviews

Main themes	Sub-categories	Evidentiary statement
Manageability	a) Normalized experience	“The nurse telephone support was crucial, because if I had a question or thought it was a weird symptom or something unusual I could talk to her’. I think the talking it out with her and realizing what I thought was maybe not manageable was manageable” (1-005). “You do not know is this how I am supposed to be feeling? … Is it normal to feel this way? So by having confirmation, by speaking and reassurance from the study nurse, it did help” (2-054)
	b) Confidence for self-care	“After talking to her, I was most confident, I felt so relieved, I feel so comfortable after talking to her”. (2-027). “After the nurse talked to me, I was so sure of myself that I was going to be just fine, that I did not have to worry about anything.” (2-033).
	c) Expert personalized advice	“I live alone. And you, here’s somebody who is concerned about you, who knows my disease, this nurse knew my disease. And, when she gave me any advice it was so helpful. It was like medicine, taking some medicine to relax, to relieve my problem. That’s so helpful, really.” (2-027) “Having somebody that you can call to say, “Is this normal, I have got this problem, what should I do, who can also give you better advice than just, you know, on the back of a package’. This is not working for me. How am I going to make this work? She gave me really good suggestions. It makes you know you can deal with it. Otherwise you feel like you are flying blind. And that’s kind of scary when you do not feel so good” (2-004).
Feeling safe	a) No need to panic	“She would assure you it was normal, so, you know, there is no need to panic over anything”. (2-040) “So it feels like you are safe because somebody’s asking about you, like somebody from the hospital is calling and saying okay. So I knew … I knew that they were all taking care of me.” (2-033)
	b) A lifeline, not on your own	“Just overall knowing that, like I said, that she was checking, that I could rely on her to call. If I had any questions or concerns that on a regular basis, I’d be talking to her.” (1-005) “I think knowing that I had a nurse call coming gave me peace of mind.” (2-056). “I could confidently talk to her about what really needed to be addressed, I guess … I found her almost like a lifeline to me some days. I just felt very confident”. (1-014)

Six nurses, two oncologists, and one pharmacist, who were actively involved in delivering the intervention, participated in end-of-study provider interviews. Content analysis of the interviews (summarized in Table 4) indicated that the providers enjoyed participating in the study and felt that the patients benefited from the intervention. Key challenges identified were fitting the additional responsibilities of the calls within existing responsibilities, documentation processes, and communication of symptom findings with the rest of the clinical team. However, the providers indicated that they thought these challenges could be overcome with appropriate tools and planning. Explicitly defining the process for identifying eligible patients, development of call tracking and documentation tools, training in symptom management for staff delivering the calls, and defining how to communicate outcome of calls were recommended by the provider participants as the main opportunities for improving implementation. There were some challenges specific to the fact that the pilot study involved both regular clinic staff and research personnel; for example, the burden of data collection was a challenge for clinic staff versus adequate experience in symptom management was seen as a potential challenge among some of the research staff.

**Table 4:** Thematic analysis of end-of-study provider interviews (*n* = 9)

Main themes	Sub-categories	Specific comments
What worked well	a) Patients liked receiving calls	• Overall the intervention was well received by patients who appreciated the personal touch the intervention added to their care. • Reassurance provided to patients regarding their treatment experience reduced anxiety. • Patients liked consistent individual performing the calls.
	b) Providers liked delivering the intervention	• Providers enjoyed their involvement. • Orientation provided to staff prior to their involvement
	c) Importance of planning	• Screening breast clinic patient lists to identify patients eligible for the study. • Determining a scheduled time to make the intervention calls to prevent missed calls. • The use of a thorough and practical tool to provide structure to the telephone calls.
What did not work	a) Fitting the intervention into existing work flow	• Providers struggled to fill calls into their existing work schedule. • Burden of large amounts of data collection and length of form to be completed during calls. • Communication between team members and incorporation of NCI PRO-CTCAE into clinic appointments. • Inconsistent staff performing intervention calls. • Patients’ confusion regarding who to call if issues arose between calls.
	b) Ensuring appropriate experience	• Clinical trial staff may not have sufficient symptom management experience compared with nurses working in the chemotherapy clinic.
Recommendations for improvement	a) Calls	• Develop a tracking tool for the telephone calls. • Timing of calls: first call for cycle one should be early. After first cycle, one call at days 5–8 may be sufficient. • Limit number of providers making follow-up calls. • Track and manage other common symptoms during calls (e.g., insomnia, anxiety, and depression).
	b) Staff training	• Further training on symptom management and organization of workload.
	c) Communication within circle of care	• Develop process to ensure oncologists and staff are aware of symptom information reported on NCI PRO-CTCAE. • Develop documentation process to ensure clear communication between team members.
	d) Other	• Reduce amount of data collection. • Improve patient symptom management guide—make it more illustrative.

### Acute care use during chemotherapy

Comparison of study participants and contemporaneous controls using administrative data (Additional file 2: Table S1) revealed that control patients were generally younger, less likely to reside in a low-income neighbourhoods, and more likely to have stage 1 disease than study participants. The types of regimens used and proportion of patients with a history of an ED visit in the year prior to their cancer diagnosis differed by centre. In regression analyses, we adjusted for age, stage, centre, and regimen. In addition, for analyses restricted to centre 2, we also adjusted for history of ED visits a year prior to cancer diagnosis. The overall adjusted incidence rate ratio for ED visits was 0.54 (95% CI 0.36–0.81) for study participants compared to controls (Table 5). While the incidence of ED visits was lower in study participants compared to controls at both centres in stratified analyses, only the results for centre 2 were statistically significant (adjusted IRR = 0.33; 95% CI 0.16–0.67). Hospitalization rates were found to be low in both groups; as such, differences were inconclusive.

**Table 5:** Crude and adjusted emergency department (ED) visits and hospitalization (H) incidence rates in study participants and contemporaneous controls by centre

	Number of patients	Total number of person-months	Emergency department visits				Hospitalizations			
			Total ED visits	IR for ED visits per month	Crude IRR (95% CI)	Adjusted IRR^a^ (95% CI)	Total H visits	IR for H visits per month	Crude IRR (95% CI)	Adjusted IRR^b^ (95% CI)
Both centres										
Control	215	860.3	257	0.30	1.00 (ref)	1.00 (ref)	59	0.07	1.00 (ref)	1.00 (ref)
AToM	77	338.7	50	0.15	0.48 (0.32, 0.73)	0.54 (0.36, 0.81)	24	0.07	1.05 (0.60, 1.85)	1.02 (0.59, 1.77)
Centre 1										
Control	145	592.5	114	0.19	1.00 (ref)	1.00 (ref)	31	0.05	1.00 (ref)	1.00 (ref)
AToM	56	233.4	32	0.14	0.72 (0.46, 1.11)	0.78 (0.51, 1.21)	11	0.05	0.90 (0.45, 1.80)	0.96 (0.48, 1.95)
Centre 2										
Control	70	267.8	143	0.53	1.00 (ref)	1.00 (ref)	28	0.10	1.00 (ref)	1.00 (ref)
AToM	21	105.3	18	0.17	0.29 (0.14, 0.62)	0.33 (0.16, 0.67)	13	0.12	1.27 (0.50, 3.19)	1.53 (0.65, 3.59)

*ED* emergency department visit, *H* hospitalization, *IR* incidence rate, *IRR* incidence rate ratio, *CI* confidence interval

^a^Regression model controlled for age, cancer stage, initial chemotherapy regimen, and centre (where applicable)

^b^Regression model controlled for age, cancer stage, initial chemotherapy regimen, history of ED use a year prior to diagnosis, and centre (where applicable)

## Discussion

Introduction of a standardized, pro-active, telephone-based toxicity management during chemotherapy for early stage breast cancer was feasible, well received by both patients and providers, and demonstrated promising preliminary results on ED utilization as compared to contemporaneous controls identified from the administrative data. Some issues related to recruitment were encountered, which were related to the availability of local resources to deliver the intervention; these can likely be overcome with changes to the implementation process, in particular, appropriate team planning and simplification of the data collection processes.

The intervention was well received by patients who, in both end-of-study surveys and interviews, indicated that they liked the support that the calls provided and especially enjoyed the relationship they developed with the nurses making the calls. The patients indicated that having real-time support to normalize the experience, and help them navigate symptoms, boosted their confidence for self-care and decreased anxiety. Nurses play a key role in supporting cancer patients throughout their illness, including beyond visits to the cancer clinic [22], but how to deliver effective remote support to patients during treatment is an area of active research. Similar to previous studies, our findings suggest that pro-active, nurse-led telephone management is a promising approach for delivering remote support between clinic visits. Whether other healthcare providers, such as oncology pharmacists [23] or clerical staff [24], could be involved in telephone support remains to be explored. While the study was well received by providers, a number of practical implementation challenges were identified that have not been addressed in previous studies, which have focused on efficacy as opposed to effectiveness of telephone-based toxicity management support.

The greatest challenges we encountered were related to identifying eligible patients, incorporating the calls into existing work responsibilities, and determining the best approach to communicate within the circle of care since the intervention nurses were often different than the usual care team. Our findings suggest that embedding pro-active symptom management into routine care requires a fundamental transformation of the “whole system”; simply adding greater expectations to existing practice systems is unlikely to be successful [25]. In a series of two randomized trials by Mooney et al. in ambulatory patients receiving chemotherapy [7, 26], the addition of a dedicated nurse to respond to symptom alerts was associated with improved symptom control, compared to relying on physicians and nurses to respond to the alerts as an add-on to their usual workload. This suggests that either creating a dedicated nursing role or ensuring protected time within an existing role is necessary for successful implementation of remote support programs. Patient identification via electronic health records (EHRs), as well as integrating call outcomes within EHRs, is an area for future study.

The incidence of ED visits during treatment in the patients that participated in the pilot was lower than that in the contemporaneous controls, although the magnitude of the effect varied by centre. While selection bias could explain this finding, telephone-based case management has been shown to decrease re-admissions in chronic diseases, such as heart failure [4, 5], and a recent single centre study of remote symptom management during chemotherapy for patients with advanced cancer also reported lower ED rates and hospitalizations in patients randomized to the intervention [13]. Furthermore, fewer acute care visits have been reported from early evaluations of the oncology patient-centred medical home whose core principles include enhanced remote support for patients receiving chemotherapy [22, 26]. We postulate that the most likely mechanism behind the lower rate of ED utilization in the setting of outpatient chemotherapy delivery is early symptom management, although our findings from patient interviews suggest that a component of benefit may be from decreasing anxiety and normalizing the experience of chemotherapy for patients with just-in-time support and standardized education.

Our study should be interpreted in the context of its limitations. We included two different centres to improve the generalizability of the findings, but the number of participating sites and sample size were limited. Furthermore, since not all patients treated at each of the centres during the intervention period were enrolled, potential for selection bias exists. While the intervention details and supporting tools were created centrally by the project steering committee, the centres were given flexibility in how they wanted to implement the intervention and data collection, and some implementation outcomes such as a recruitment rate were not reliably captured. This highlights the importance of conducting formal pilot studies prior to large-scale implementation trials. Involvement of research personnel facilitated patient identification and data collection, but involvement of clinical personnel is essential for sustainability and spread beyond the research setting which is the cornerstone of implementation science. Figuring out the optimal balance between research and clinical personnel in these types of studies requires additional work. Finally, due to the resource limitations, symptom management calls were not recorded or analysed for content; the quality of the calls could have varied by provider. Although our findings suggest that pro-active symptom management may help to optimize care in ambulatory cancer population, larger scale evaluations are needed to determine impact and sustainability. Selecting patients at higher risk of toxicity or focusing the intervention on high-risk periods, such as early in the chemotherapy course or when there is a change in drug, may help to optimize resource use and facilitate scalability of the intervention [27, 28]; however, a validated prediction model to identify high-risk patients does not currently exist [29]. Further analysis using administrative data and retrospective chart review could help to identify high-risk patients and high-risk periods for intervention.

## Conclusions

In summary, our study suggests that pro-active, telephone-based toxicity management during chemotherapy in routine care is feasible, perceived as valuable by patients and providers and may have a positive impact on acute care utilization. Larger-scale evaluations of the impact of this approach on acute care utilization and patient-reported outcomes are warranted, but attention to implementation issues needs to be considered prior to initiation. We are currently conducting a 20-centre cluster-randomized trial of this intervention (NCT02485678).

## Additional files

Additional file 1: **Figure S1.** Selection of contemporaneous controls in administrative data. (PDF 30 kb)

Additional file 2: **Table S1.** Baseline demographic and clinical characteristics of selected controls and study participants from administrative data, by centre. (DOCX 19 kb)
