# Supplementary figures and images for: Ambulatory Toxicity Management (AToM) Pilot: results of a pilot study of a pro-active, telephone-based intervention to improve toxicity management during chemotherapy for breast cancer
Source: Pilot Feasibility Stud. 2019 Mar 8;5:39. doi: 10.1186/s40814-019-0404-y (PMC6407231; doi:10.1186/s40814-019-0404-y)

**Supplementary Figure 1.**

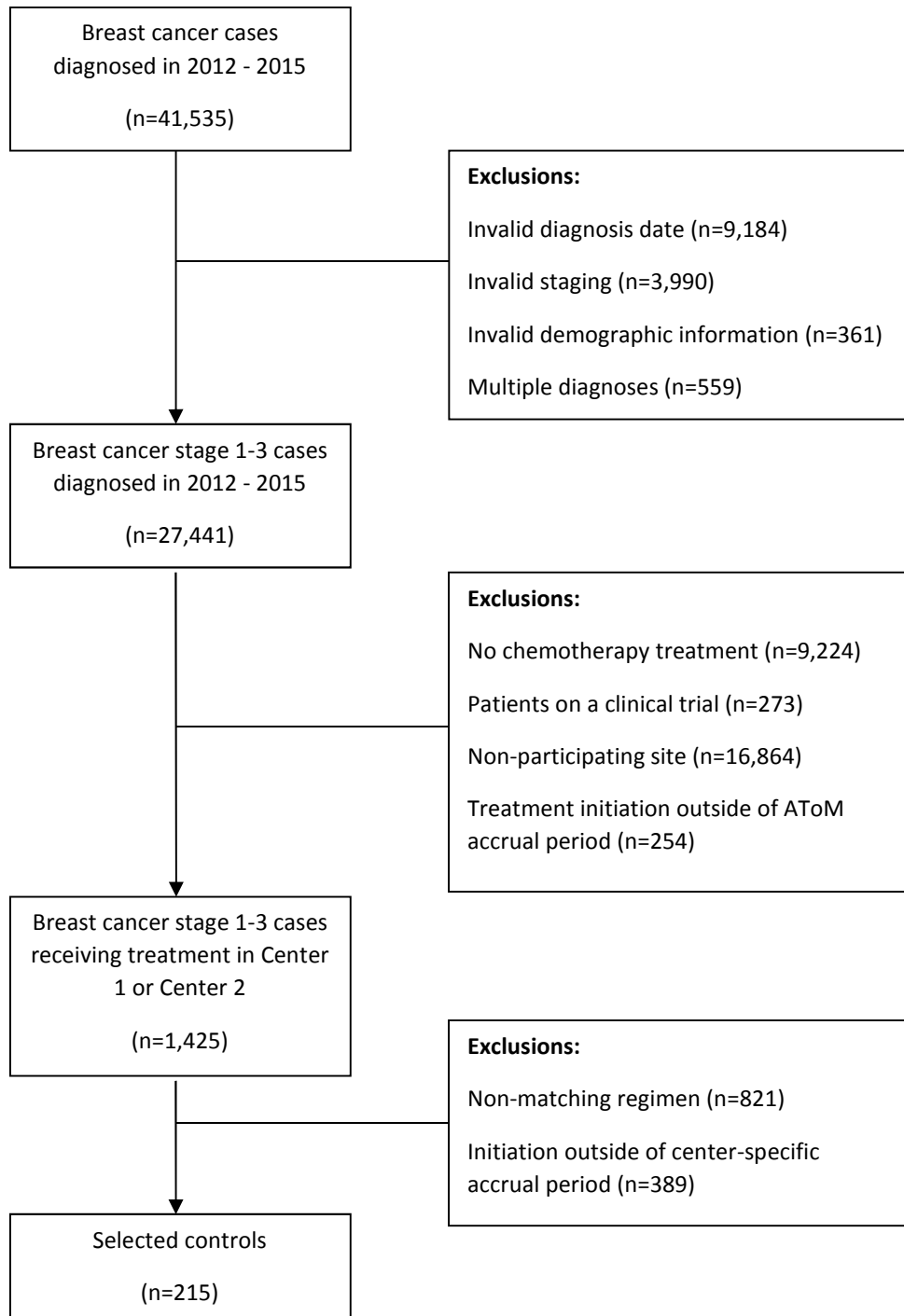

Supplement: Supplementary file 1 — Figure S1. Selection of contemporaneous controls in administrative data. (PDF 30 kb) [file 40814_2019_404_MOESM1_ESM.pdf]
