# Supplementary material for: Ambulatory Toxicity Management (AToM) Pilot: results of a pilot study of a pro-active, telephone-based intervention to improve toxicity management during chemotherapy for breast cancer
Source: Pilot Feasibility Stud. 2019 Mar 8;5:39. doi: 10.1186/s40814-019-0404-y (PMC6407231; doi:10.1186/s40814-019-0404-y)
Supplement: Supplementary file 2 — Table S1. Baseline demographic and clinical characteristics of selected controls and study participants from administrative data, by centre. (DOCX 19 kb) [file 40814_2019_404_MOESM2_ESM.docx]

**Table S1** Baseline demographic and clinical characteristics of selected controls and study participants from administrative data, by center.

|  | | | **Center 1** | | | **Center 2** | | |
| --- | --- | --- | --- | --- | --- | --- | --- | --- |
| **Variable** | | | **Control** | **AToM** | **Standardized difference (%)^a^** | **Control** | **AToM** | **Standardized difference (%)^a^** |
|  |  |  | **n=145** | **n=56** |  | **n=70** | **n=21** |  |
| **Age at diagnosis, mean (SD)** | | | 50.0 (11.3) | 53.6 (10.9) | -32.4 | 57.3 (10.5) | 58.5 (11.0) | -11.3 |
| **Neighbourhood Income Quintile, n (%)** | | |  |  |  |  |  |  |
|  | Lowest Quintile | | 14 (9.7) | 11 (19.6) | -28.5 | <6 | 6 (28.6) | -58.3 |
|  | Medium to Low Quintile | | 16 (11.0) | 6 (10.7) | 1.0 | 9 (12.9) | * | 28.9 |
|  | Middle Quintile | | 34 (23.4) | 12 (21.4) | 4.8 | 17 (24.3) | * | 12.7 |
|  | Medium to High Quintile | | 37 (25.5) | 10 (17.9) | 18.7 | 15 (21.4) | * | 18.7 |
|  | Highest Quintile | | 42 (29.0) | 17 (30.4) | -3.0 | 22 (31.4) | 7 (33.3) | -4.1 |
|  | Unknown | | 2 (1.4) | 0 |  | ** | 0 |  |
| **Stage at diagnosis, n (%)** | | |  | | |  | | |
|  | | Stage I | 25 (17.2) | 7 (12.5) | 13.4 | 16 (22.9) | * | 9.4 |
|  | | Stage II | 79 (54.5) | 30 (53.6) | 1.8 | 41 (58.6) | 13 (61.9) | -6.8 |
|  | | Stage III | 41 (28.3) | 19 (33.9) | -12.2 | 13 (18.6) | ** | -1.2 |
| **Regimen categories, n (%)** | | |  |  |  |  |  |  |
|  | | AC-P weekly | 0 | 0 |  | 21 (30.0) | 7 (33.3) | -7.2 |
|  | | AC-P Q14D | 64 (44.1) | 30 (53.6) | -19.0 | 20 (28.6) | * | 22.5 |
|  | | AC-P Q21D | 0 | 0 |  | 14 - 19 | * | -2.3 |
|  | | FEC-100 | 0 | 0 |  | 10 - 15 | * | -12.8 |
|  | | FEC-T | 57 (39.3) | 15 (26.8) | 26.9 | 0 | 0 |  |
|  | | TC | 19-23 | 6-10 | -5.3 | 0 | 0 |  |
|  | | Other | * | * | -9.9 | 0 | 0 |  |
| **Number of chemotherapy administrations, mean (SD)** | | | 6.5 (2.3) | 6.7 (2.1) | -11.7 | 7.2 (5.4) | 9.4 (4.7) | -45.1 |
| **Charlson comorbidity score, mean (SD)** | | | 0.0 (0.1) | 0.0 (0.0) | 16.7 | 0.1 (0.4) | 0.2 (0.5) | -38.9 |
| **At least one hospitalization one year prior to cancer diagnosis, n (%)** | | | 10 (6.9) | * | 25.3 | * | * | -20.8 |
| **At least one ED visit one year prior to cancer diagnosis, n (%)** | | | 24 (16.6) | 8 (14.3) | 6.3 | 23 (32.9) | * | 44.8 |

SD = standard deviation; AC-P = Adriamycin, cyclophosphamide and paclitaxel; FEC-100 = 5-fluorouracil, epirubicin and cyclophosphamide; FECT-T = 5-fluorouracil, epirubicin, cyclophosphamide and docetaxel; TC = docetaxel and cyclophosphamide; ED = emergency department.

^a^ Standardized difference in excess of 10% may indicate meaningful covariate imbalance between control and AToM intervention groups.

**Data suppressed due to small sample size
